# MicroRNA in Control of Gene Expression: An Overview of Nuclear Functions

**DOI:** 10.3390/ijms17101712

**Published:** 2016-10-13

**Authors:** Caterina Catalanotto, Carlo Cogoni, Giuseppe Zardo

**Affiliations:** Department of Cellular Biotechnologies and Hematology, University of Rome Sapienza, Rome 00179, Italy

**Keywords:** microRNA, miRNA inducing silencing complex (miRISC), transcriptional control, epigenetics, nuclear function

## Abstract

The finding that small non-coding RNAs (ncRNAs) are able to control gene expression in a sequence specific manner has had a massive impact on biology. Recent improvements in high throughput sequencing and computational prediction methods have allowed the discovery and classification of several types of ncRNAs. Based on their precursor structures, biogenesis pathways and modes of action, ncRNAs are classified as small interfering RNAs (siRNAs), microRNAs (miRNAs), PIWI-interacting RNAs (piRNAs), endogenous small interfering RNAs (endo-siRNAs or esiRNAs), promoter associate RNAs (pRNAs), small nucleolar RNAs (snoRNAs) and sno-derived RNAs. Among these, miRNAs appear as important cytoplasmic regulators of gene expression. miRNAs act as post-transcriptional regulators of their messenger RNA (mRNA) targets via mRNA degradation and/or translational repression. However, it is becoming evident that miRNAs also have specific nuclear functions. Among these, the most studied and debated activity is the miRNA-guided transcriptional control of gene expression. Although available data detail quite precisely the effectors of this activity, the mechanisms by which miRNAs identify their gene targets to control transcription are still a matter of debate. Here, we focus on nuclear functions of miRNAs and on alternative mechanisms of target recognition, at the promoter lavel, by miRNAs in carrying out transcriptional gene silencing.

## 1. Biogenesis of miRNAs

The most studied class of small non-coding RNAs (ncRNA) are the miRNAs. They are encoded within the genomes of organisms ranging from plants to animals. It has been estimated that they are able to modulate up to 60% of protein-coding genes in the human genome at the translational level [1]. Because of their ubiquitous role in gene regulation, they are involved in many physiological processes, such as differentiation, proliferation, apoptosis and development, and their dysregulation has been related to various pathological disorders, including muscular dystrophy [2], diabetes [3] and several types of cancer [4].

Biogenesis of miRNAs occurs through a multi-step process requiring both a nuclear and a cytoplasmic phase. They are transcribed typically by RNA polymerases II or III as long primary miRNA (pri-miRNA) with a cap and a poly-A tail. pri-miRNAs contain a double-stranded stem of about 30 base pairs, a terminal loop and two flanking unstructured single-stranded tails. pri-miRNAs are processed into short 70-nt stem-loop structures known as precursor miRNAs (pre-miRNAs) by a protein complex, the Microprocessor complex, which consists of the RNase III enzyme Drosha, and a double stranded-RNA binding protein, Di George syndrome critical region 8 gene (DGCR8). 

Further processing of pre-miRNAs by the RNase III enzyme Dicer generates miRNA duplexes (ds-miRNAs) [5,6] with a phosphate at the 5′ end and a 2-nt overhang with a hydroxyl at the 3′ end [7,8,9]. The miRNA duplex, is successively loaded onto Argonaute (AGO) itself by an RNA inducing silencing complex (RISC) comprising Dicer, *trans*-activation response RNA-binding protein (TRBP) and AGO. TRBP identifies the “guide” and the “passenger” strands in the ds-miRNA molecule. TRBP senses the thermodynamic properties of the ds-miRNAs, and once it recognizes the strand with the less stable 5′ end, the protein loads the ds-miRNA in the correct orientation onto AGO proteins. AGO unwinds the duplex, and removes the passenger strand retaining the mature miRNA molecule [5,10]. A subclass of miRNAs, called mirtrons, bypass the initial Drosha cleavage and instead depend on splicing and debranching to produce the pre-miRNA molecules which are the substrates for Dicer mediated miRNA maturation [11,12]. Another subset of miRNAs originating in short introns, agotrons, has been recently characterized [13]. Mature agotrons are similar to pre-miRNAs (80–100 nt), and their biogenesis is independent of both Drosha and Dicer although they are ultimately associated and stabilized by Argonaute (AGO) proteins [14]. pre-miRNAs are translocated to the cytoplasm in a process mediated by Exportin5 (XPO5), a member of the karyopherin β family, in a complex with Ran·GTPase [15].

## 2. Function of Cytoplasmic miRNAs

In plants, and rarely in mammals, perfect complementarity between miRNA and target mRNA induces the cleavage and disruption of the latter [16]. In animals, seminal studies on miRNAs have shown that only the seed region (sequence spanning from position 2 to 8 at the 5′ end), is crucial for target recognition. The seed sequence pairs fully to its responsive element mainly at the 3′ untranslated region (UTR) of the target mRNA [17]. However, miRNA responsive elements (MREs) are also ubiquitously widespread in 5′ UTR sequences as well as in coding regions of mRNAs [18,19,20]. Moreover, recent advances in the transcriptome-wide method of mapping miRNA binding sites (AGO HITS-CLIP) demonstrated that a number of miRNA-target interactions in vivo are mediated through non-canonical seed pairing rules (~15%–80%) [21,22].

The cornerstone of the miRNA functioning is the formation of the RISC composing minimally of an AGO protein and a miRNA molecule. The utility of this binary complex is highlighted by the following factors: (i) miRNAs can specifically recognize their mRNA targets through Watson and Crick base pairing, and at the same time, may easily detach and reattach to different targets, thus exerting their control serially on a wide range of target molecules; (ii) the AGO proteins provide an extremely efficient platform onto which modulating cofactors can bind their targets. Whereas miRNA is responsible for recognizing the mRNA target to be modulated, the task of AGO proteins is to determine the mode of action of the RISC-mediated gene regulation. Despite their high sequence similarity and the fact that critical aminoacids are conserved also in other AGO proteins, only AGO2 possesses endonuclease activity, if the miRNA-mRNA base pairing is perfect [23,24]. It is, therefore, still uncertain what the exact functions of the different AGO proteins are. 

The endonucleolytic ability to degrade cellular mRNAs is not frequently adopted by mammalian RISCs. Most often, the minimal RISC (AGO2 and miRNA) acts as a platform to recruit proteins that define the fate of the RISC’s target mRNAs [24,25,26]. These factors have been grouped according to their biological functions as: proteins required for RISC assembly, proteins involved in translational regulation, messenger RNA-binding proteins and proteins involved in RNA metabolism. Therefore, in addition to those factors closely required to build a functional minimal RISC (i.e., Dicer, TRBP and Hsp90 proteins, allowing incorporation of the miRNAs into the RISC) a large group of AGO interacting proteins is necessary to explain the mechanism of RISC mediated gene regulation in animals.

## 3. miRNA-RISC: Cytoplasmic Activity

Since its discovery 20 years ago, the primary function of the miRNA-RISC (miRISC) appeared to be post-transcriptional mRNA regulation in the cytoplasm. Several models have been proposed to explain the mechanism used by the miRNA-RISC complex to control mRNA fate. In general, miRISCs either hinder translation or enhance mRNA degradation. For a long time, the predominant idea was that variation in the composition of RISCs led to alternative mechanisms for post-transcriptional control. More recently, the outlook has changed. The current model proposes that, in a sequential process, the RISC switches off its targets by repressing mRNA translation at first and subsequently initiating degradation [27]. However, it has been suggested that the sequential order of these two events reflects kinetic differences between translational repression and mRNA decay rather than a cause-and-effect relationship between them [27,28,29].

Ribosome-profiling experiments [30] and polysome profiling combined with high-throughput sequencing methods ruled out that miRISC post-transcriptional control occurs through inhibition of translation elongation, protein degradation or ribosome drop-off. In addition, Bazzini et al. [29] found that miR-430 reduces the number of ribosomes on target mRNAs in wild type and Dicer mutant zebrafish embryos indicating that miRNAs can regulate translation at an initiation step. Removal of the poly(A) tail did not affect miRISC dependent translational repression suggesting that miRISC induced deadenylation represents a level of miRNA mediated control independent from translation. The opposite is also true; miRNA guided translational repression is not required for miRNA dependent deadenylation [31]. Béthune et al. [32] provided further support for these observations, showing that translational repression is the dominant effect on newly synthesized targets, whereas mRNA deadenylation and decay are prevalent at steady state.

However, in quantitative terms, destabilization of target mRNAs is the main cause for reduced protein output. In fact, translational repression provides roughly 6%–25% of the global repression in protein expression [33,34]. In recent years, the CCR4-NOT complex has been identified as the main effector downstream of the RISC complex. The CCR4–NOT complex coordinates mRNA deadenylation directly and indirectly, by recruiting PABP proteins or DEAD box helicases, such as eIF1A and DDX6, mRNA decapping and translational repression. Thus, several authors have explored the details of the mechanistic events and protein effectors regulating miRNA mediated target decay and translational repression shedding light on miRISC activity in the cytoplasm (for a review see Jonas and Izaurralde [35]).

## 4. miRISC and miRNAs: Cytoplasmic/Nuclear Shuttling

Within the last few years, there have been several reports indicating that miRISC complexes are also present in mammalian cell nuclei. Western blots of nuclear fractions, cell fractionation and immunofluorescence have revealed the presence of AGO proteins, Dicer, TRBP, and Gw182/TNRC6 in cell nuclei [36,37,38,39], where they combine to form multi-proteins complexes [40]. By performing protein fractionation and ammonium sulphate precipitation assays on HeLa nuclear and cytoplasmic extracts, Gagnon and co-workers [40] suggested that the composition of miRISC complexes and binding stability of their components differ in the nucleus relative to the cytoplasm. At the same time, they found that the nuclear Dicer programmed with exogenous 21-nt siRNAs retains its catalytic activity as well as AGO2, and is able to target nuclear RNAs, such as 7SK small nucleolar RNAs (snoRNA), inducing its degradation [40]. However, the loading process appeared to occur in the cytoplasm, despite the simultaneous presence of Dicer and TRBP within the nucleus. The current hypothesis is that the miRNA-AGO2 assembly occurs outside the nucleus, where some critical loading factors are present. Once established, the minimal RISC may subsequently be imported into the nucleus. This assumption is in accordance with previous analysis suggesting that the nuclear RISC seems to be smaller than its cytoplasmic counterpart [37]. More recently, using a semi quantitative mass spectrometry approach, Kalantari et al. [41] have confirmed the small size of the nuclear RISC, showing that the association of AGO2 with Gw182/TNRC6 (-A, -B, -C) and AGO3 is best preserved and more stable in both nuclei and cytoplasm whereas the AGO2 interaction with Dicer and TRBP is confined to the cytoplasm. Obviously, different conditions for biochemical purification may yield diverse compositions of the RISC. 

These observations overall suggest that the minimal RISC might constitute the core component of a larger machinery designed to modulate gene expression also in the nucleus. However, the mechanism guiding the cytoplasmic-nuclear shuttling of miRISC remains an open question. A family of proteins, called karyopherins, mediates the translocation of the RISC into the nucleus. These proteins recognize nuclear localization sequences in the protein cargoes and then assist their active transport through the nuclear pore complex. In detail, the AGO-miRNA complex is imported into the nucleus by binding to importin 8 (IPO8) [38,42]. In fact, silencing of IPO8 reduces the pool of nuclear AGO2 [38]. AGO2 is lacking a nuclear localization signal unlike many components of the cytoplasmic RISC, including TNRC6 paralogs and Dicer [39,40,41,42,43], although a recent paper questions Dicer nuclear compartmentalization [44]. However, AGO2 overcomes this deficiency by combining with TNRC6A which possesses a nuclear localization signal (NLS) rather than a nuclear export signal (NES), and works as a nuclear–cytoplasmic shuttling protein to re-localize minimal miRISC to the nucleus [39,45]. It is possible that IPO8, TNRC6A and AGO2 localize together in cytoplasmic P-bodies where they form a final complex capable of binding to nuclear pore complex (NPC) entering the nucleus. On the other hand, the NES element of TNRC6A affects AGO2 nuclear export. Indeed, inhibition of exportin1 (XPO1) results in nuclear accumulation of both of these proteins [39,46]. 

The basic idea is that the RISC can undertake more than one route to reach the nuclear compartment. In parallel with the identification of nuclear RISCs, miRNAs have been detected in the nuclear compartment through microarray analysis and small RNA deep sequencing of nuclear extracts [47,48,49,50]. Gagnon and co-workers [40] have found that about 75% of whole cell miRNAs are present in both the nucleus and the cytoplasm. They also demonstrated that the relative abundance of miRNAs in the cytoplasm reflects their relative proportion in the nucleus also, suggesting an inability of the miRNA cytoplasmic-nuclear shuttling mechanisms to internalize specific miRNAs. Several studies have shown that nuclear miRNAs co-localize with TNRC6A [39], which is a main component of cytoplasmic miRISC. This finding has given rise to the idea that miRNAs can be relocated to the nucleus through miRISC shuttling. 

In contrast to the hypothesis of an unselective distribution of miRNAs, several studies have suggested the existence of a motif-dependent mechanism that selectively translocates miRNAs into the nucleus. For instance, a seminal study reported that a hexanucleotide element (AGUGUU) at the 3′ end of miR-29b is responsible for its selective nuclear localization in HeLa cells. Indeed, translocation of this 6-nt motif to other small RNAs is able to induce their nuclear accumulation [51]. Subsequent work has partially confirmed the importance of the 3′-terminal sequences in defining cellular localization of mature miRNAs. In addition, it has been suggested that the miRNAs containing a 3′ ASUS element (where S may be a cytosine or guanosine) or a 3′-terminal guanine nucleotide, which can be added in a non-template manner, are preferentially localized within the nucleus [49,50]. Therefore, it is possible that machinery involved in a motif-dependent miRNA nuclear translocation, able to grant nuclear access of specific miRNAs, exists. However, data from different laboratories suggest that whatever the selective system of miRNA nuclear translocation is, it may be differentially expressed in various cell types. Indeed, the pool of miRNAs enriched in the nuclei appears to be cell line specific. It is also useful to consider some data demonstrating that exogenous siRNAs are preferentially able to localize to the cytoplasm or to the nucleus in a manner dependent on the trapping activity of their targets [52,53]. They move back and forth between nucleus and cytoplasm, and they are retained in the cellular compartment where they identify their target molecules.

## 5. miRNAs: Nuclear Functions

Some results demonstrate that miRNA may have different nuclear functions, in some cases, independent of RISC activity. Actually, only a limited number of miRNAs are representative of these non-canonical roles, and little is known about their mode of action. 

### 5.1. Regulation of RNA Transcriptome

miRISCs were found to induce post-transcriptional degradation of small RNA molecules, such as long non-coding RNAs confined to the nucleus. For example, the nuclear miR-709 seems to inhibit miRNA15a/16-1 maturation by binding to a 19-nt recognition element on pri-miR-15a/16-1 and thus blocking its processing into pre-miR-15a/16-1 in mouse cells [54]. In a similar manner, the mature let-7 miRNA in *Caenorhabditis elegans* physically associates with its own pri-miRNA within the nucleus promoting the processing of the pri-miRNA transcript, and thus providing a positive feedback loop [55]. 

An unbiased analysis of miRNA-mRNA-Argonaute interactions in mouse brain using high-throughput sequencing of RNAs isolated by cross-linking immunoprecipitation [56] showed that 4% of AGO-mRNA tags mapped to long non coding RNAs (lncRNAs). LncRNAs are >200 bases long with low or no protein coding potential. These RNAs often regulate epigenetic silencing of genes through chromatin remodeling. Several lines of evidences suggest that lncRNA expression levels may undergo miRNA-guided regulation into the nucleus. In this view, specific AGO-miRNA complexes seem able to target miRNA-complementary sequences within lncRNA affecting their stability and function [57,58,59,60,61]. Thus, these results reveal a possible new nuclear function of miRNAs: miRNAs might contribute to the regulation of the non-coding RNA transcriptome. 

### 5.2. Nucleolar Function

miRNAs have been found to be significantly concentrated in the nucleolus as both pre-miRNAs and mature miRNAs [62,63]. Various hypotheses have been proposed in order to describe possible biological functions of nucleolar miRNAs [63,64]. For instance, the fact that miR-206 co-localizes with 28S ribosomal RNA (rRNA), both in the nucleolus and the cytoplasm in mammalian cells, may suggest that miRNA can associate with ribosome subunits at an early stage of ribosome biogenesis [65]. As shown by Atwood et al. [66], minimal RISC (AGO2 and miRNA) may affect rRNA abundance in cells. Moreover, the binding of minimal RISC to the 45S rRNA seems to be sensitive to Dicer knockdown and actinomycin D treatment suggesting that tethering of AGO2 on rRNA depends on the presence of miRNAs and at the same time requires an ongoing Pol I activity. Based on these findings, it is possible to speculate that miRNA-targeted rRNAs may confer onto the mature ribosomes some specific properties that help to define their interaction with accessory proteins. Reyes-Gutierrez et al. [67] have also proposed that miRNAs bind to some mRNA targets in the nucleolus before their export to the cytoplasm where mRNAs arrive in a pre-silenced status. Alternatively, nucleoli may constitute the site of storage for miRNAs, which are redistributed into the nucleoplasm and/or cytoplasm following genotoxic stress [64], thus evoking the ancestral role of the RISC as a genome defence system.

Furthermore, it has been suggested that pri- and pre-miRNAs may be A-to-I edited in the nucleolus, as several RNA editing enzymes, such as the adenosine deaminases (ADARs), accumulate in the nucleolus. This modification would inhibit maturation of miRNAs and thus reduce the cellular availability of mature miRNAs [68], even though no edited miRNAs have been identified in the pool of nucleolar miRNA to date [62,65,69,70]. All together, the above-mentioned models connect miRNAs with nucleoli and indicate that nucleolar compartmentalization of miRNAs is required to affect mRNA post-transcriptional regulation in the cytoplasm. Thus, dynamic movement of miRNAs to and from the nucleolus is part of the program of mRNA regulation, which ultimately ends in the cytoplasm. 

### 5.3. Regulation of Alternative Splicing

An emerging idea is of a hypothetical coordination between miRNA-mediated gene control and splicing events in gene regulatory networks. Several studies have suggested that maturation of specific miRNAs may depend on splicing factors [71,72]. Immunoprecipitation assays for nuclear AGO1 and AGO2 proteins have highlighted their interaction with core components of the splicing machinery and several splicing factors [73] underlining the interdependence between the two pathways. In order to define both nuclear AGO2 and miRNA target sites on a transcriptome-wide scale, crosslinking immunoprecipitation assays coupled with high throughput sequencing (HITS-CLIP) analyses have been used. Through these assays, AGO2 and miRNA binding sites were identified within intronic sequences, at a frequency ranging from 12%–15% in brain samples of mouse and human, respectively, and up to 3% in human myocardial cells [56,74,75]. These results constitute an important starting point to support the idea of miRNA involvement in the cellular splicing program. However, it cannot be excluded at this time that the isolated sequences represent retained intronic tracts targeted by RISC in the cytoplasm [74]. Future work will be necessary to answer this question as well as to identify specific miRNAs able to modulate alternative splicing. 

Functional studies have in fact demonstrated the capability of AGO proteins to redirect splicing only in association with exogenous small RNAs [76,77] or with endogenous small RNAs that are different from miRNAs [73]. Data obtained from these studies are not in complete agreement and depict two possible modes of action to explain miRISC participation to splicing modulation. Alló et al. [76] revealed that small duplex RNAs, targeting both intronic and exonic sequences, near alternative exons, could favor the inclusion of the variant exon. This process, dependent on AGO1, is associated with an increase in H3K9me2 and H3K27me3 and is sensitive to Heterochromatin Protein 1 (HP1) knockdown, suggesting a *modus operandi* of miRISC similar to that proposed for miRNA-guided transcriptional gene silencing. More recently, Alló et al. [78] also found that ~80% of nuclear AGO1 clusters associate with transcriptional and non-transcriptional enhancers and thus influence alternative and constitutive splicing more than transcription of the neighbouring genes. Similar conclusions were reached by Ameyas-Zazoua et al. [73]. They showed that AGO1 and AGO2 are required to induce remodelling of chromatin structure and splicing efficiency. To evaluate the impact of endogenous small RNA depletion on alternative splicing events, both Alló and Ameryas-Zazoua groups have tested the effect of Dicer knockout on splicing outcome. Data suggest that Dicer-dependent small RNAs, such as endogenous small RNAs and miRNAs, are required to maintain the cellular splicing pattern. Moreover, the two studies are in agreement in proposing an antisense transcript, rather than pre-mRNA, as RISC-mediated splicing control target. In contrast, Liu et al. [77,79] have suggested that both duplexes, RNA molecules and single stranded RNAs complementary to sequences near exon/intron junctions, may modulate splicing by leading AGO2 to pre-mRNAs. These small oligonucleotides would induce exon skipping in a chromatin modification independent manner. The proposed model means that RISC binds the nascent transcript and thus impairs recruitment of the spliceosomal complex to the splice junctions without affecting pre-mRNA transcription levels and stability. 

### 5.4. Transcriptional Role of miRNAs

We know that siRNAs can regulate gene transcription [80], thus derogating from the most consolidated activity of RNA interference. The fact that miRNAs possess a size comparable to that of siRNAs, together with the idea that miRNAs and RISC elements are present in the nucleus, led to examination of their ability to regulate gene expression at the transcriptional level. An important number of studies have shown that miRNAs may mediate transcriptional gene activation (TGA) or transcriptional gene silencing (TGS), thereby broadening the spectrum of activities of miRNAs and revealing that miRNAs may function not only at the post-transcriptional level. The first miRNA identified as an activator of gene transcription (TGA) was human miR-373, which induced transcription of both E-cadherin (*CDH1*) and cold-shock domain-containing protein 2 (*CSDC2*) [81]. A discrete number of reports have subsequently confirmed this observation. Other miRNAs have also been identified as activators of gene transcription suggesting the existence of a new and widespread mechanism of miRNA guided gene expression regulation [82,83,84,85,86,87]. These studies have led to the identification of the conditions required for miRNA guided TGA but not the biochemistry of this process or the relationship between protein effectors. A requirement for TGA is the presence in gene promoters, of a DNA sequence complementary to the 5′-seed region in the miRNA and partially to the flanking region. However, whether full or partial complementarity of the seed region is a strong determinant for TGA remains unclear. 

**Figure 1:**
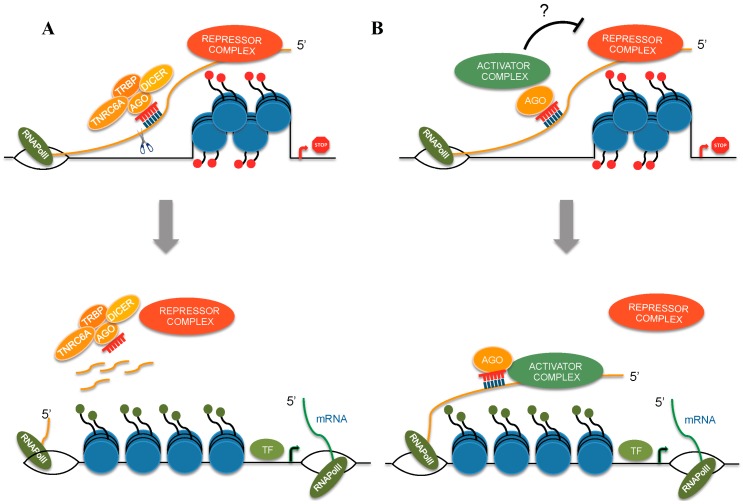
Examples of alternative mechanisms of miRNA guided transcriptional gene activation (TGA). (**A**) Long non coding RNAs (lncRNAs) or promoter associated RNAs (pRNAs) can silence gene expression by recruiting a transcriptional repressive complex. miRNAs targeting a complementary sequence within the lncRNA or pRNA would recruit a nuclear RISC and induce cleavage of the antisense lncRNA or pRNA transcript promoting exclusion of repressive complex and derepression of the sense transcription; (**B**) Alternatively, miRNAs induce gene activation by recruiting a protein complex consisting of transcriptional activators. TNRC6A, Trinucleotide repeat-containing 6A; TRBP, *trans*-activation response RNA-binding protein; AGO, Argonaute; TF, Transcription Factor.

Recruitment of AGO proteins (mainly AGO2) [83] and RNApolII enrichment [83,85] at the regulated gene promoters as well as modification of chromatin marks (increase of activator marks as H3K4me3) accompany miRNA TGA [83]. As previously mentioned, several components of miRISC have been identified in the human nucleus. Furthermore, a miRISC does exist in the nucleus and is characterized by a diverse complexity compared to the cytoplasmic one. AGO proteins, Dicer1, TARBP2 and Gw182/TNRC6, have been identified in the nucleus and seem essential for mediating transcriptional roles of miRNAs [36,37,39,40,88,89,90,91]. Although the mechanism or sequence of events by which miRNAs induce TGA, are far from completely understood, several hypotheses have been proposed based on the available data. One possible mechanism lies in the observation of the pervasion of bidirectional transcription in the human genome. The human genome is transcribed in the both sense and antisense directions [92,93,94,95]. Although the role of bidirectional transcription is not clear, we know that it originates in non-coding antisense transcripts (mainly lncRNAs) overlapping sense promoters. The role of these non-coding transcripts would be to repress the transcription of adjacent genes by recruitment of repressive complexes, which mainly consist of chromatin effectors and modifiers in close proximity of targeted gene promoters. The recognition by the miRNA seed region of a perfectly matching sequence within a non-coding promoter transcript would recruit a nuclear RISC and induce cleavage of the antisense non-coding transcript, removal of chromatin effectors and derepression of the corresponding sense transcription [96,97] (Figure 1A). An alternative mechanism is that the miRNA seed sequence recognizes a complementary sequence in the 5′ UTR promoter region of a nascent RNA or in a non-coding promoter transcript (pRNA). In this case, the minimal miRISC (AGO-miR) would recruit a protein complex to a gene promoter region, consisting of proteins and activating chromatin modifiers able to shift the chromatin structure toward a more permissive arrangement and thereby increase gene transcription levels [85] (Figure 1B). This recruitment hypothesis is supported by the observation that miRNA TGA is independent of target RNA cleavage [85].

On the other hand, miRNAs have also been found to induce TGS. The first report describing miRNA induced TGS was by Kim et al. [98]. Kim et al. described the transcriptional silencing activity of miR-320. This miRNA, encoded within the promoter region of the *POLR3D* gene in the antisense orientation, directed *POLR3D* transcriptional inhibition through recruitment of AGO1, Polycomb group (PcG) component EZH2 and chromatin changes as observed in tri-methyl histone H3 lysine 27 (H3K27me3) repressive mark enrichment. Numerous other examples of miRNAs inhibiting transcription of a single gene or of small group of genes have subsequently been reported [99,100,101,102]. In addition, we and other authors have shown that miRNA driven transcriptional repression may affect general cellular activities, such as senescence, nerve regeneration and granulopoiesis [91,103,104]. 

**Figure 2:**
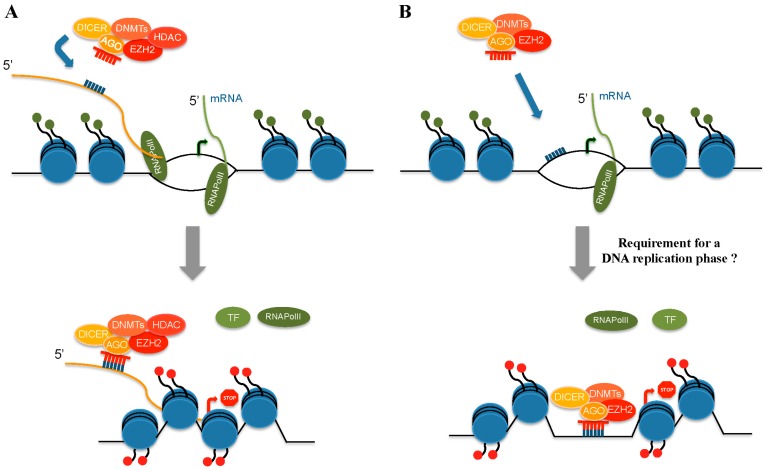
Examples of alternative mechanisms of miRNA guided transcriptional gene silencing (TGS). miRNAs can inhibit gene expression at transcriptional level. (**A**) In this case, miRNA-targeted non-coding promoter associated RNA would represent a docking platform for a protein inhibitory complex consisting of elements of RISC, PcG proteins and chromatin modulators. This interaction would enable a protein inhibitor complex to be in close proximity of the targeted promoter region, the chromatin structure of which would be modified to establish a non-permissive transcriptional status; (**B**) Alternatively, miRNA guided recognition and interaction with promoter regions may occur through a direct interaction between RNA and single-stranded DNA complementary sequences. DNMTs, DNA methyltransferases; HDAC, Histone deacetylase; AGO, Argonaute; EZH2, Enhancer of zeste homolog 2; Transcriptional Factor, TF; RNA Polymerase II, RNApolII.

It is surprising that both activities (TGA and TGS) require similar features, components and effectors, suggesting that these two apparently opposing biological events are tightly functionally related. For instance, it is in general agreement that miRNA TGS requires the existence of promoter-overlapping antisense transcription, the presence of miRNA seed-matches in the antisense transcribed strand, and recruitment of AGO1 and/or AGO2 protein at the gene promoter, but differs from TGA in the release of RNAPolII. In TGS, epigenetic modifications have a more prominent role than in TGA. Recruitment of PcG proteins, modification of H3K27me3 (increase) and H3K4me3 (decrease) levels, and DNA methylation are frequently observed at miRNA regulated promoter regions. Although the protein effectors of miRNA TGS have been identified, the nature of the recognition mechanism and interaction between miRNAs and targeted promoters remains unclear. A number of studies suggest a direct interaction between the miRNA seed sequence and the complementary sequence in the transcribed strand, mainly in the antisense orientation, of the promoter overlapping the promoter itself. In this view, the miRNA-targeted non-coding promoter associated RNA (pRNA) would represent a docking platform for a protein inhibitory complex consisting of elements from the RISC (AGO and Dicer1 proteins), PcG elements (YY1, EZH2 and SUZ12), and chromatin modifiers. This interaction would enable a protein inhibitor complex to be in close proximity of the targeted promoter region, the chromatin structure of which would be modified to establish a non-permissive transcriptional status (Figure 2A). This model is based on the evidence from transcriptome studies revealing that over 70% of gene promoters are overlapped by non-coding RNA transcripts [105] and that gene promoter sequences are enriched with potential miRNA target sites [106] in both sense and antisense orientations. Experimental procedures using synthetic gapmers or siRNA approaches to inactivate pRNA function support results from the genomic analysis [89,95,96,97].

However, it is well known that small RNAs are capable of hybridizing not only with other RNAs, but also with double-stranded DNA to form relatively stable RNA*DNA:DNA triplexes via Hoogsteen or reversed Hoogsteen base-pairing [107,108,109,110]. Therefore, alternative mechanisms of miRNA directed TGS should be investigated and taken into consideration. The first evidence of transcriptional regulation via the formation of a RNA*DNA:DNA triplex structure derives from the observations of Martianov et al. [111]. These investigators observed RNA-dependent repression of the major dihydrofolate reductase gene (*DHFR*) by a non-coding regulatory transcript transcribed from the upstream minor promoter of this gene. This non-coding RNA induced promoter-specific transcriptional repression through the disruption of the formation of the pre-initiation complex at the major DHFR promoter. In order to demonstrate specificity of the RNA-dependent repression, Martianov et al. [111] investigated the properties of the major DHFR promoter. This promoter was found to be rich in GC content and contained several G-track sequences. Such sequences had been previously shown to form stable purine-purine-pyrimidine triplex structures (H form) between DNA and RNA. Thus, they analyzed the formation of the stable triplex structure between the major DHFR promoter and the regulatory transcript. A standard H-form band-shift assay revealed the formation of a specific DNA–RNA complex. Interestingly, a small synthetic oligoribonucleotide, corresponding to the core of the major promoter, recapitulated the effects of the regulatory transcript and formed a stable H form with promoter DNA, suggesting that small RNAs (such as miRNAs) have the potential to target double stranded DNA. 

The existence of transcription-regulating RNA*DNA:DNA structures is further supported by work performed by Schmitz et al. on the mechanism of transcriptional repression of silent *rRNA* genes [112]. These *rRNA* genes are silenced by NoRC, which requires association with pRNA for its function. This pRNA originates from an RNA polymerase I promoter located upstream of the pre-rRNA transcription start site. NoRC function depends on the interaction with the hairpin-forming sequence of the pRNA. However, ectopically expressed pRNA lacking this sequence was able to induce DNA hypermethylation and gene silencing as well as the full-length pRNA. Ectopic expression of different truncated forms of pRNA revealed that transcriptional repression was induced as long as the RNA sequence matching the T_0_ site (the binding site for the transcription factor TTF-1) on rDNA was conserved. Thus, a stretch of 20-nt matching the T_0_ DNA sequence was sufficient to guarantee the transcriptional repression of silent *rRNA* genes. In addition, in in vitro mobility shift and DNA protection assays, the possible formation of a triplex structure formed by small stretch of 20-nt in the pRNA and the T_0_ DNA sequence was observed. These studies along with the observation that triple-helix target sites were found to be overrepresented at human gene promoters [113,114], provide innovative insights depicting alternative mechanisms of action of small ncRNA (including miRNAs) in TGS. However, the in vivo existence of RNA*DNA:DNA triplexes and their role still need to be validated. 

Alternatively, the observation that small RNAs can interact directly with the double-stranded promoter DNA yields a second possibility, that RNA guided recognition and interaction with promoter regions may occur through a direct interaction between RNA and single-stranded DNA complementary sequences. This view was proposed by Jacob and Monod who argued that base complementarity would enable RNA to interact specifically with other nucleic acid sequences in order to establish and transmit diverse activities, including transcriptional activation, inhibition, recruitment of protein effectors, and chromatin modification, at specific loci. Some experimental data substantiates a model based on the recognition and direct interaction between small RNAs and complementary DNA sequences at gene promoters. Regarding TGA, Zhang et al. [113] have reported that some cellular miRNAs in human peripheral blood mononuclear cells associate with RNApolII and the TATA binding protein. They also were found to bind directly to TATA box motifs at gene promoters, inducing transcription of the target gene. In addition, Nakama et al. [114], through RNA immunoprecipitation assays, showed that an ncRNA, transcribed from both strands of centromeric *dh* repeats in fission yeast, was associated with chromatin through the formation of a DNA-RNA hybrid. The presence of DNA-RNA hybrids in the cell was confirmed by immunofluorescence analysis with an anti-DNA-RNA hybrid antibody. Overexpression or depletion of RNase H in in vivo experiments led to decreased and increased levels, respectively, in the amount of the DNA-RNA hybrid that formed, and in both experiments, heterochromatin changes were induced.

Important assessments can also be made from studies specifically evaluating the mechanism of miRNA guided TGS. For instance, we demonstrated that miR-223 drives TGS of *NFI-A* through two DNA sequences within the *NFI-A* promoter region complementary to the miR-223 seed sequence [91]. We demonstrated through multiple approaches that during human granulopoiesis, miR-223 entered the nucleus and localized at its complementary sequences on the *NFI-A* promoter. These approaches included confocal microscopy of transfected fluorescent miR-mimics and in situ hybridization to the endogenous miRNA with fluorescent oligonucleotides complementary to the sequence of miR-223. We also detected miR-223-specific signals on mitotic chromosomal spreads from cells undergoing myeloid differentiation. These signals were detectable on symmetrical regions of sister chromatids, suggesting a gene-specific localization. Since, RNA synthesis is blocked during metaphase, this finding may support a view whereby miR-223 directly binds to DNA on specific sequences. By combining transfection of a Cy5-labeled miR-223 with anti-Cy5 chromatin immunoprecipitation, we were able to demonstrate that miR-223 does interact with the *NFI-A* promoter DNA. Here, miR-223 is part of a ribonucleoprotein repressive complex involving YY1, Dicer1 and AGO1. The localization of this complex on the *NFIA* promoter was RNA-dependent, since it was prevented by RNase H which specifically recognizes RNA:DNA hybrids. In addition, in experiments with stable cell lines engineered to express a promoter-luciferase cassette including ~1.4 kb of the *NFI-A* promoter, the integrity of the two miR-223 DNA sequences complementary to the miR-223 seed was found to be necessary for transcriptional inhibition. Our data suggest that miR-223-mediated TGS may occur by a mechanism that does not rely on antisense transcription, as it does not occur when reporter constructs are used. Based on our observations, however, we instead prefer a mechanism that requires an RNA:DNA interaction (Figure 2B). In addition, although Martianov et al. [111] favor a model based on antisense-promoter-transcription guidance, they cannot exclude a direct interaction between RNA and DNA to explain RNA-dependent repression of *DHFR*. In a similar manner, Adilakshmi et al. [104] suggested that miR-709 exerted its effect by binding in *trans* and regulating the antisense strand of the *EGR2* promoter because the Myelin specific element (MSE) exhibits miRNA binding sites in both strands. However, the same authors also state “It is beyond doubt however, that miR-709 interacts directly with the MSE-promoter DNA as indicated by the susceptibility of the complex to RNase H treatment”. More revealing, are data generated by Miao et al. [102] demonstrating that miR-552 transcriptional repression was mediated by the binding, through its non-seed sequence, to a hairpin loop in the DNA cruciform structure in the *CYP2E1* promoter region.

## 6. Conclusions

**Figure 3:**
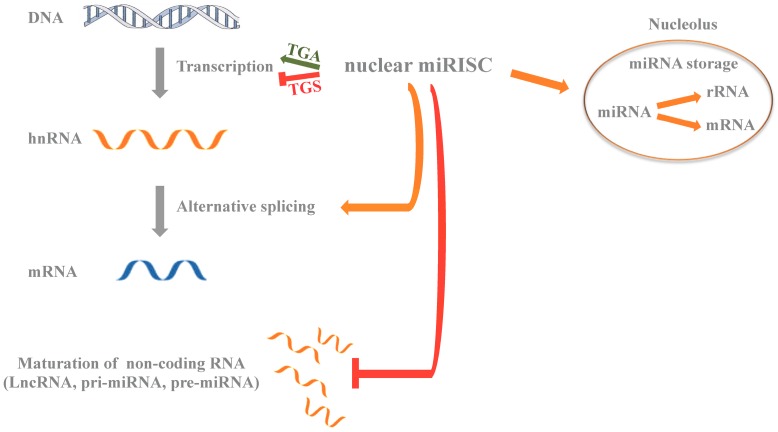
Schematic representation of the function of miRNAs in the nucleus. Nuclear miRNAs may activate (TGA) or inhibit (TGS) gene transcription, block maturation of non-coding RNAs (LncRNA, pri-miRNA, pre-miRNA), affect alternative splicing (orange arrow); more controversial is miRNAs function in the nucleolus where they might affect maturation of ribosomal RNAs (rRNAs) and of messenger RNA (mRNAs) (orange arrow). pri-miRNA, primary miRNA; pre-miRNA, precursor miRNAs; miRISC, miRNA inducing silencing complex.

Taken together, these data demonstrate that there is tremendous versatility in miRNA function. miRNAs have clear roles in both the cytoplasm and nucleus where they have been found to regulate gene expression through different mechanisms. In the nucleus (Figure 3), emerging data implicates miRNAs in the regulation of the stability of mRNA in nucleoli and alternative splicing. More compelling data points to direct involvement in the regulation of gene expression at the transcriptional level. Here, miRNAs may mediate both activation and inhibition of transcription of a target gene. Both of these biological processes appear to be dependent on the presence of a DNA promoter sequence complementary to the seed region of a regulatory miRNA and on a non-coding transcript overlapping the gene promoter itself. The recognition between the miRNA and the complementary sequence on the non-coding RNA occurs through classic Watson and Crick base pairing. Although this model is currently the most widely favored, there is data suggesting that miRNA guided recognition and interaction with promoter regions may occur through a direct interaction between RNA and single-stranded DNA complementary sequences. More evidence is needed to support this mechanism of action of miRNAs in vivo, but this alternative model is attractive because of its simplicity and high specificity. miRNAs therefore might bind directly and specifically to promoter DNA, just as transcription factors, and serve as a platform for the localization of transcriptional regulators and chromatin modifying proteins at genomic loci.

The versatility and complexity of mechanisms by which miRNAs regulate gene expression, at cytoplasmic and nuclear level, imply that alteration of miRNAs regulatory mechanisms may affect normal cellular functions. The alteration of miRNAs biogenesis mechanism, miRNAs expression level and miRNAs regulatory networks affects important biological pathways such as cellular differentiation and apoptosis and it is detected in various human diseases and syndromes, especially in cancer (for a review see Ghai and Wang [115]). All tumors present specific signatures of miRNAs altered expression. For this reason, miRNAs expression profiles of tumors may represent valid and useful biomarkers for diagnosis, prognosis, patient stratification, definition of risk groups and to monitor the response to therapy. In most cases these profiles are obtained from biopsy material. The biggest advantage of using miRNAs is that they are released from the tumor tissue in the plasma where they can be easily collected and analyzed, especially if embedded in extracellular vesicles [115]. To date miRNAs represent the most promising class of molecular biomarkers. Equally relevant is the emerging role of microRNAs in viral infections. Data from literature show a mutual interference between viruses and the host cell’s miRNA machinery. For instance, viruses may impair the host cell’s miRNA pathway by interacting with specific proteins; synthesize their own miRNAs to modify cellular environment or to regulate their own mRNAs; or make use of cellular miRNAs to their favor. However, it is also true that host cell’s miRNAs may target viral mRNAs. In many cases, this bidirectional interference is resolved in favor of the viruses that as a result may escape the immune response and complete the replication cycle (for a review see Verma et al [116] and Piedade and Azevedo-Pereira [117]).
